# Droplet digital PCR-based nucleic acid amplification test for improved molecular and clinical diagnosis of severe fever with thrombocytopenia syndrome patients

**DOI:** 10.1128/jcm.00367-26

**Published:** 2026-07-27

**Authors:** Yujin Baek, Minchae Koo, Yong Shin, Chang-Seop Lee, Il-Hwan Kim

**Affiliations:** 1 Division of Biomedical Metrology, Korea Research Institute of Standards and Science 65408, Daejeon, South Korea; 2 Department of Biotechnology, College of Life Science and Biotechnology, Yonsei University 26721, Seoul, South Korea; 3 Department of Biological Sciences, Korea Advanced Institute of Science and Technology 34968, Daejeon, South Korea; 4 Department of Internal Medicine, Jeonbuk National University Medical School 90158, Jeonju, South Korea; 5 Research Institute of Clinical Medicine of Jeonbuk National University-Biomedical Research Institute of Jeonbuk National University Hospital 65377, Jeonju, South Korea; 6 Department of Precision Measurement Science, University of Science and Technology, Daejeon, South Korea; Mayo Clinic Minnesota, Rochester, Minnesota, USA

**Keywords:** severe fever with thrombocytopenia syndrome, molecular diagnosis, droplet digital PCR, clinical diagnosis, tick-borne zoonosis

## Abstract

Severe fever with thrombocytopenia syndrome (SFTS) is caused by SFTS virus (SFTSV) and is characterized by high mortality and non-specific clinical manifestations, which make early and accurate molecular diagnosis essential. This study aims to investigate whether reverse transcription droplet digital PCR (RT-ddPCR) provides improved diagnosis of SFTS compared to existing molecular diagnostic platforms. Detection limits of RT-ddPCR, reverse transcription quantitative PCR (RT-qPCR), and reverse transcription nested PCR (RT-nested PCR) were determined using RNA extracted from metrologically validated SFTSV nucleic acid reference material. Then, diagnostic performance metrics of each PCR method were compared using RNA from 29 SFTS patients and 29 disease controls with scrub typhus. Statistical analyses of key laboratory parameters were performed to identify markers significantly correlated with SFTSV load, as defined by RNA copy number, in early SFTS patients. RT-ddPCR showed the lowest detection limit of 3.82 copies/μL among the three PCR methods. The sensitivity of RT-ddPCR was 82.75% in clinical samples, which was higher than that of RT-qPCR at 72.41%. The specificity of RT-ddPCR was 100% compared to 89.66% for RT-nested PCR. Overall, RT-ddPCR demonstrated the highest diagnostic performance among the three PCR methods, with an accuracy of 91.38% and an area under the curve value of 0.91. A strong correlation between viral RNA loads and laboratory parameters was observed in the early stage of SFTS, particularly with high-sensitivity C-reactive protein, aspartate aminotransferase, alanine aminotransferase, lactate dehydrogenase, and activated partial thromboplastin time. Our findings demonstrate that a novel molecular diagnosis method using RT-ddPCR shows improved diagnostic performance for detecting SFTSV RNA from SFTS patients and potentially contributes to accurate early diagnosis and enhanced clinical diagnosis.

**IMPORTANCE:**

Severe fever with thrombocytopenia syndrome (SFTS) is a high-mortality tick-borne infection caused by SFTS virus (SFTSV), and reliable molecular diagnosis is essential for patient care in endemic regions. Existing PCR-based assays for SFTS show limited sensitivity and specificity, particularly in patients with suspected or early-stage disease. Here, we describe the development and analytical validation of a reverse transcription droplet digital PCR (RT-ddPCR) assay for the detection and quantification of SFTSV RNA from patient sera. By using metrologically validated viral nucleic acid reference materials and clinical samples, this assay demonstrates significantly improved sensitivity and overall diagnostic performance compared with conventional reverse transcription quantitative PCR and reverse transcription nested PCR. RT-ddPCR can provide clinical microbiology laboratories with a useful tool to enhance early and reliable molecular diagnosis of SFTS.

## INTRODUCTION

Severe fever with thrombocytopenia syndrome (SFTS) is a hemorrhagic fever disease caused by infection of SFTS virus (SFTSV), which is transmitted while ticks feed on human hosts (1). SFTS patients are found in East Asian countries, including China, Japan, and Korea, with high mortality rates ranging from 5% to 35%, depending on identified regions (2). Due to the lack of an approved preventive vaccine or targeted antiviral treatment for SFTS, accurate and reliable early diagnosis is important to reduce the current high mortality rates (3).

Molecular diagnostic technologies are considered the gold standard for diagnosis of infectious diseases due to their high specificity and sensitivity compared to serological or other methods (4, 5). Molecular diagnosis of SFTS is commonly performed by detection of SFTSV RNA through nucleic acid amplification by PCR-based methods, such as reverse transcription quantitative PCR (RT-qPCR) and reverse transcription nested PCR (RT-nested PCR) (6). RT-qPCR offers high specificity, but its sensitivity decreases at lower viral RNA concentrations (7, 8), and this limitation may lead to false-negative results in patients with suspected SFTS, especially during the early stages of infection (9). RT-nested PCR is more sensitive than RT-qPCR and is widely used for diagnosis of SFTS patients in the early stages of infection (10). However, nested PCR is susceptible to cross-contamination and carry-over contamination because the first-round PCR tubes must be opened and the amplified products handled before the second round of amplification, which can lead to false-positive results (11). Moreover, RT-nested PCR does not provide quantitative information on viral RNA in patient samples; therefore, it is difficult to define disease prognosis based on RT-nested PCR test results (12).

Droplet digital PCR (ddPCR), the third-generation PCR system, has been reported to offer higher sensitivity compared to RT-qPCR and RT-nested PCR (13, 14). In the ddPCR system, the reaction mixture is first partitioned into a large number of nanoliter-sized droplets; PCR amplification proceeds to endpoint within each droplet; and fluorescence is then measured for each droplet individually rather than in a single bulk reaction (15). Consequently, reverse transcription droplet digital PCR (RT-ddPCR) allows for direct quantification of target RNA molecules, unlike RT-qPCR or RT-nested PCR (16). Therefore, it is worth investigating whether RT-ddPCR is more sensitive and specific than that of RT-qPCR and RT-nested PCR for detecting RNA from SFTS patient sera. In addition, since RT-ddPCR can quantitate RNA copy numbers of SFTSV from patient samples, correlation between SFTSV RNA copy numbers and key laboratory parameters from patient samples should be examined for identifying clinical markers that are directly associated with viral RNA loads, especially for suspected or early-stage patients.

Here, we developed a novel universal molecular diagnostic assay for SFTS, which targets conserved regions across six prevalent genotypes of SFTSV. We then quantitatively compared the detection limits among RT-ddPCR, RT-qPCR, and RT-nested PCR using metrologically certified SFTSV nucleic acid reference material. Subsequently, we used 29 individual SFTS-positive and negative patient sera to evaluate the diagnostic performance of RT-ddPCR by comprehensively comparing the sensitivity, specificity, accuracy, receiver operating characteristic (ROC) curves and area under the curve (AUC) of each molecular diagnostic system. Lastly, we analyzed the correlation between viral RNA copy numbers and key laboratory parameters of SFTS. Our results reported the first quantitative comparison among three PCR methods for diagnosing SFTS and confirmed the improved molecular and clinical diagnosis of SFTS using RT-ddPCR.

## MATERIALS AND METHODS

### Patient selection and sample preparation

This study included a total of 58 adult patients, consisting of 29 patients diagnosed with SFTS and 29 febrile control patients. Each specimen was obtained from a unique, individual patient. Specimens were collected at various timepoints during hospital management, rather than strictly at the time of initial diagnosis, with intervals from symptom onset ranging from 2 to 33 days (see Table 2). All SFTS patients were consecutively enrolled after admission to a tertiary care hospital in South Korea between May 2018 and November 2021. SFTS was confirmed exclusively by the detection of SFTSV RNA using reverse transcription polymerase chain reaction (RT-PCR). The control group consisted exclusively of patients with scrub typhus. In South Korea, scrub typhus and SFTS represent the two most prevalent vector-borne infectious diseases and frequently occur during overlapping seasonal periods. Because these diseases share similar clinical presentations, including acute febrile illness, thrombocytopenia, and elevated liver enzymes, differentiation between scrub typhus and SFTS is a critical diagnostic challenge in endemic regions. Therefore, patients with scrub typhus were selected as the disease control group to evaluate the diagnostic specificity and clinical utility of molecular assays for SFTS under real-world conditions. Patients aged ≥18 years who were clinically suspected of having scrub typhus were eligible for inclusion in the control group. Laboratory confirmation of scrub typhus was based on at least one of the following criteria: (i) an indirect immunofluorescence assay (IFA) IgG titer ≥1:256 against *Orientia tsutsugamushi*; (ii) a ≥4-fold increase in IFA antibody titer in paired serum samples; or (iii) a positive nested PCR result targeting the 56 kDa type-specific antigen gene of *O.* (17).

### Droplet digital PCR, quantitative PCR, and nested PCR experiments

Viral RNA was extracted from human serum reference material spiked with SFTSV nucleic acid reference material or patient sera. The RT-ddPCR, RT-qPCR, and RT-nested PCR experiments were then performed to detect and quantify SFTSV viral RNA. An in-house pan-genotypic primer and probe set targeting SFTSV medium (M) segment was designed for nucleic acid amplification tests (Table S1). The detection limit, sensitivity, specificity, accuracy, and AUC were calculated based on amplification results to evaluate and compare the diagnostic performance of the three PCR systems.

### Statistical analysis

Statistical analysis of patient samples was conducted using SPSS software (v23; IBM Corporation, USA). Fisher’s exact test and the Mann–Whitney *U* test were used to compare categorical and continuous variables between SFTS and ST patient groups. The probit analysis for limit of detection (LoD) was conducted using MedCalc software (v19.2.1; MedCalc Software, Belgium). Spearman’s rank correlation coefficient was calculated to assess the correlation between SFTSV RNA copy number and laboratory parameters from the early-stage SFTS patient subgroup. All statistical analyses and illustrations were generated using GraphPad Prism 10 (GraphPad Software, USA). A *P* value of <0.05 was considered statistically significant.

## RESULTS

### Patient information

In this study, baseline clinical and laboratory characteristics were compared between SFTS-positive patients and an SFTS-negative control group. The SFTS-negative group consisted of 29 patients diagnosed with scrub typhus. Within this control group, the diagnosis was confirmed by PCR in 23 patients (79.3%) and by IFA in 16 patients (55.2%), with 8 patients (27.6%) demonstrating a fourfold or greater rise in IgG antibody titers.

Demographically, SFTS-positive patients were older, with a mean age of 73.4 years compared to 69.1 years in the SFTS-negative group, although this difference was not statistically significant (*P* = 0.164) (Table 1). Female patients were less prevalent in the SFTS-positive group (58.6%) compared to the SFTS-negative group (79.3%, *P* = 0.178) (Table 1).

**TABLE 1:** Demographics, clinical characteristics, and laboratory findings of SFTS patients and control^*a*^

Characteristics	Positive (*n* = 29)	Negative (*n* = 29)	*P* value
Epidemiology, no. (%)			
Age, mean Y ± SD (range)	73.4 ± 9.7	69.1 ± 12.9	0.164
Female	17 (58.6)	23 (79.3)	0.178
Comorbidities, no. (**%**)			
Cardiovascular disease^*b*^	1 (3.4)	3 (10.3)	0.352
Cerebrovascular disease	5 (17.2)	1 (3.4)	0.423
Pulmonary disease^*c*^	1 (3.4)	1 (3.4)	1.000
Diabetes mellitus	6 (20.7)	10 (34.5)	0.258
Malignancies	1 (3.4)	4 (13.8)	0.423
Clinical signs and symptoms, no. (**%**)			
Headache	2 (6.9)	12 (41.4)	0.003
Dyspepsia	12 (41.4)	10 (34.5)	0.602
Nausea/vomiting	9 (31.0)	10 (34.5)	1.000
Abdominal pain	6 (20.7)	4 (13.8)	0.731
Chills	19 (65.5)	20 (69.0)	1.000
Myalgia	16 (55.2)	14 (48.3)	0.803
Fatigue	18 (62.1)	19 (65.5)	1.000
Rash/eschar	4 (13.8)	28 (96.6)	<0.001
Tick bite	7 (24.1)	27 (93.1)	<0.001
Laboratory values, median (IQR)			
WBC count (×1,000/mm^3^)	1.60 (1.29–2.07)	7.1 (4.51–8.63)	<0.001
Platelet count (×1,000/mm^3^)	67.0 (35.0–98.0)	124 (93.0–149.0)	<0.001
aPTT (s)	38.6 (33.9–45.2)	31.8 (27.9–33.6)	0.184
Total bilirubin (mg/dL)	0.5 (0.35–0.60)	0.7 (0.5–1.0)	0.048
Albumin (g/dL)	3.7 (3.4–4.1)	3.7 (3.3–3.9)	0.320
AST (IU/L)	142 (65–655)	92 (51–145)	0.243
ALT (IU/L)	69 (34–149)	68.0 (32–101)	0.239
LDH (IU/L)	2,021 (682–2,260)	590 (495–989.5)	0.002
Creatinine (mg/dL)	1.0 (0.68–1.22)	0.8 (0.64–0.99)	0.866
hs-CRP, mg/L	4.4 (0.74–10.19)	91.0 (56.5–150.5)	<0.001
SFTS genotype, no. (**%**)			
SFTS type M segment			
Type A	5 (17.3)		
Type B1	8 (27.6)		
Type B2	7 (24.2)		
Type B3	6 (20.7)		
Type C	1 (3.4)		
Type D	1 (3.4)		
Type F	1 (3.4)		
Scrub typhus diagnostic method, no. (**%**)^*d*^			
		PCR positive	23 (79.3)
		Serology (IFA IgG)	16 (55.2)
		Fourfold increase	8 (27.6)

^*a*^ ALT, alanine aminotransferase; aPTT, activated partial thromboplastin time; AST, aspartate aminotransferase; CRP, C-reactive protein; IQR, interquartile range; LDH, lactate dehydrogenase; SD, standard deviation; SFTS, severe fever with thrombocytopenia syndrome; WBC, white blood cell; Y, years.

^*b*^ Includes myocardial infarction, congestive heart failure, and peripheral vascular disease.

^*c*^ Chronic obstructive pulmonary disease and asthma.

^*d*^ Total percentages may exceed 100% as some patients were confirmed by both methods.

Laboratory findings revealed that SFTS-positive patients had significantly lower white blood cell (WBC) counts (median 1.60 × 1,000/μL vs 7.1 × 1,000/μL) and platelet counts (median 67 × 1,000/μL vs 124 × 1,000/μL), alongside significantly elevated lactate dehydrogenase (LDH) levels (median 2,021 IU/L vs 590 IU/L) compared to the SFTS-negative group (all *P* < 0.001) (Table 1). Additionally, total bilirubin levels were slightly lower in SFTS-positive patients (median 0.5 mg/dL vs 0.7 mg/dL, *P* = 0.048), while high-sensitivity C-reactive protein (hs-CRP) levels were substantially lower in the SFTS-positive group (median 4.4 mg/L vs 91.0 mg/L, *P* < 0.001) (Table 1).

Finally, genotypic analysis of the SFTS virus within the positive group revealed B1 (27.6%) and B2 (24.2%) as the most common types, followed by B3 (20.7%) and type A (17.3%) (Table 1).

### Detection limit comparison among droplet digital PCR, quantitative PCR and nested PCR using SFTSV nucleic acid reference material

Multiple sequence alignment results for the SFTSV M segment target region confirmed that the primer–probe binding sites used in this study are highly conserved across genotypes A–F (Fig. S1). Serial dilution analysis of viral RNA from all six genotypes using the in-house universal assay showed consistent detection with high linearity (*R*^2^ ≥0.99), which indicates comparable quantitative performance across genotypes (Fig. S2). Taken together, these results demonstrate that the in-house primer–probe set can detect all six SFTSV genotypes using RT-ddPCR or RT-qPCR (Fig. S3 and S4). RT-nested PCR also generated amplified products for all six genotypes using the pan-genotypic first and second PCR primer sets (Fig. S5). Thus, we confirmed that detection limit measurements were conducted using primers and probes capable of detecting all six SFTSV genotypes in each of the three PCR methods.

**Fig 1:**
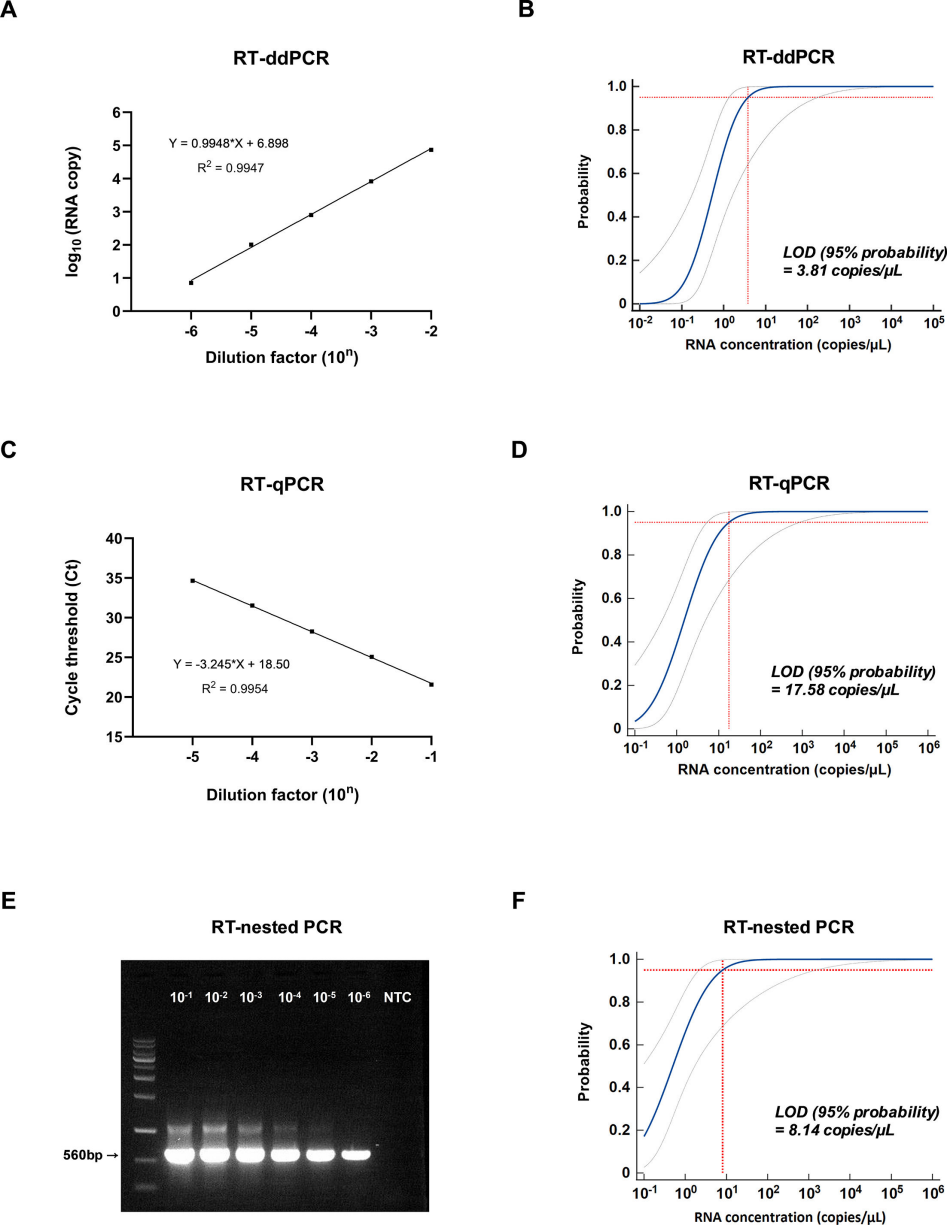
Evaluation of detection limits for RT-ddPCR, RT-qPCR, and RT-nested PCR using SFTSV nucleic acid reference material spiked into human serum. (**A**) Linear regression analysis of RT-ddPCR for SFTSV nucleic acid reference material. All experiments were performed in triplicate, with data presented as means ± SEM. (**B**) Probit analysis sigmoid curve reporting the LoD of RT-ddPCR. The red dashed line represents the RNA concentration corresponding to 95% probability (copies/μL). (**C**) Standard curve of RT-qPCR for SFTSV RNA reference material. (**D**) Probit analysis sigmoid curve reporting the LoD of RT-qPCR. (**E**) The secondary nested PCR products amplified from the SFTSV RNA reference material showing the expected product size of 560 bp. (**F**) Probit analysis sigmoid curve reporting the LoD of RT-nested PCR.

SFTSV nucleic acid reference material was used to compare the detection limits of RT-ddPCR, RT-qPCR, and RT-nested PCR (Fig. 1). To evaluate detection limits under conditions resembling actual clinical samples, the reference material was spiked into human serum and serially diluted 10-fold from 1.27 × 10^5^ to 1.27 × 10^−1^ copies/μL. RT-ddPCR detected SFTSV RNA at concentrations as low as 1.27 copies/μL (Fig. 1A). Probit regression analysis of eight replicate reactions per concentration determined the LoD of RT-ddPCR at a 95% detection probability to be 3.82 copies/μL (Fig. 1B; Fig. S6A). In contrast, RT-qPCR consistently showed amplification signals at concentrations of 12.7 copies/μL and above (Fig. 1C), and its LoD was 17.58 copies/μL (Fig. 1D; Fig. 6B). RT-nested PCR detected SFTSV RNA down to 1.27 copies/μL as well (Fig. 1E), whereas its LoD was calculated to be 8.14 copies/μL (Fig. 1F; Fig. S6C). These results demonstrate that RT-ddPCR had enhanced detection performance in both the detectable range and the statistically defined LoD.

### Improved molecular diagnostic performance in SFTS patient samples using droplet digital PCR system

The diagnostic metrics of RT-ddPCR, RT-nested PCR and RT-qPCR were calculated by nucleic acid amplification results of SFTSV RNA from 29 SFTS-positive and 29 SFTS-negative patients (Tables 2 and 3 and Fig. S7 to S9). RT-ddPCR successfully detected and quantified viral RNA from patient sera as low as 4.66 copies/μL (Table 2).

**TABLE 2:** Detection and quantification of SFTSV RNA in clinical specimens from SFTS patients using RT-ddPCR, RT-qPCR, and RT-nested PCR

Sample no.	Diagnosis	RT-ddPCR (copies/μL)	RT-qPCR (Ct)	RT-nested PCR (+/−)	Time from symptom onset to specimen collection
1	SFTS	TMTC^*a*^	18.99	+	4
2	SFTS	TMTC	20.87	+	10
3	SFTS	395,838.97	23.24	+	3
4	SFTS	2.37	39.55	−	12
5	SFTS	2.43	39.19	+	8
6	SFTS	0	38.29	+	33
7	SFTS	138.5	34.92	+	8
8	SFTS	4.81	36.88	+	9
9	SFTS	1,241.87	32.14	+	15
10	SFTS	1,087.13	32.04	+	11
11	SFTS	3,248.87	30.27	+	9
12	SFTS	2,380.94	31.01	+	12
13	SFTS	79,953.19	25.79	+	7
14	SFTS	161.11	34.72	+	10
15	SFTS	1,172.08	31.99	+	9
16	SFTS	86,526.43	26.01	+	2
17	SFTS	234,312.36	23.56	+	5
18	SFTS	4,304.83	29.57	+	6
19	SFTS	438.6	32.97	+	5
20	SFTS	31,001.86	29.71	+	7
21	SFTS	1,205.5	31.84	+	6
22	SFTS	0	39.85	−	12
23	SFTS	75,440.90	25.53	+	7
24	SFTS	TMTC	16.18	+	10
25	SFTS	29,412.26	27.16	+	4
26	SFTS	4.7	38.72	−	25
27	SFTS	4.66	38.85	+	9
28	SFTS	16.24	38.57	−	24
29	SFTS	0	39.83	+	19

^*a*^ TMTC, too many to count. Values above the upper limit of quantitation of the RT‑ddPCR assay (i.e., >1.27 × 10^4^ copies/μL) were considered above the quantitative range and reported as TMTC. Positive/negative classification was based on the following cutoffs: RT‑ddPCR, ≥3.82 copies/μL (95% LOD); RT‑qPCR, Ct ≤35; RT‑nested PCR, presence of a visible band of the expected size in the second‑round PCR.

**TABLE 3:** Detection and quantification of SFTSV RNA in clinical specimens from febrile patients using RT-ddPCR, RT-qPCR, and RT-nested PCR^*a*^

Sample no.	Diagnosis	RT-ddPCR (copies/μL)	RT-qPCR (Ct)	RT-nested PCR (+/−)
1	ST	0	N/A	−
2	ST	2.57	42.36	−
3	ST	0	N/A	−
4	ST	0	41.77	−
5	ST	2.49	N/A	−
6	ST	0	38.21	−
7	ST	0	38.50	−
8	ST	0	N/A	−
9	ST	0	N/A	−
10	ST	0	N/A	−
11	ST	2.42	39.58	−
12	ST	0	42.45	−
13	ST	0	38.43	−
14	ST	0	N/A	−
15	ST	0	40.71	−
16	ST	0	39.31	−
17	ST	0	N/A	−
18	ST	0	39.71	−
19	ST	0	40.08	−
20	ST	0	N/A	−
21	ST	0	39.37	−
22	ST	0	40.33	−
23	ST	0	39.69	−
24	ST	0	N/A	−
25	ST	0	39.26	+
26	ST	0	N/A	−
27	ST	0	37.74	+
28	ST	0	43.97	−
29	ST	0	37.76	+

^*a*^ Positive/negative classification was based on the following cutoffs: RT‑ddPCR, ≥3.82 copies/μL (95% LOD); RT‑qPCR, Ct ≤35; RT‑nested PCR, presence of a visible band of the expected size in the second‑round PCR. N/A, not available (no amplification).

**Fig 2:**
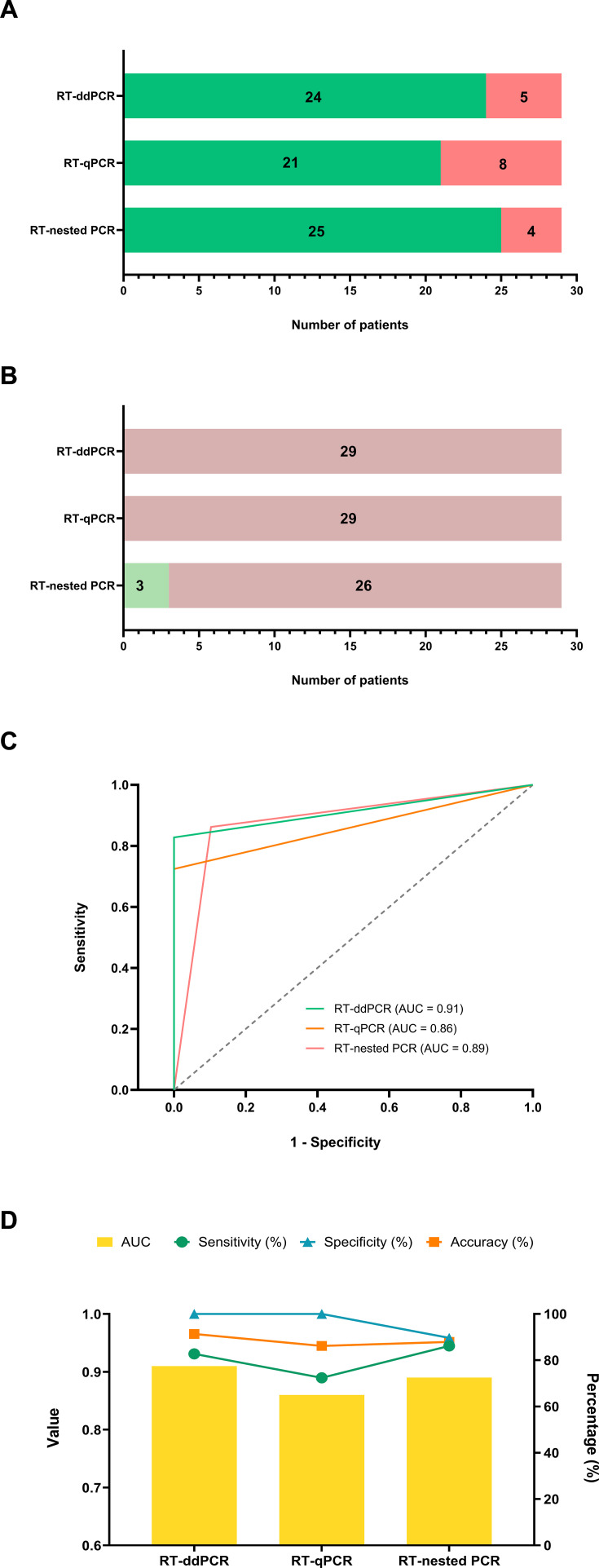
Detection of SFTSV and evaluation of diagnostic performance in clinical samples across the three PCR methods. (**A**) Detection results in clinical samples from SFTS patients. Green bars represent positive results, and red bars represent negative results, with patient counts displayed on each bar. (**B**) Detection results in clinical samples from febrile patients. Light green bars represent positive results, and light red bars represent negative results, with patient counts displayed on each bar. (**C**) Receiver operating characteristic curves and area under the curve (AUC) values for RT-ddPCR, RT-qPCR, and RT-nested PCR. (**D**) Summary of diagnostic performance metrics, including AUC, sensitivity, specificity, and accuracy for each PCR method.

In contrast, RT-qPCR failed to detect viral RNA copy numbers below 16.24 copies/μL, which was defined by RT-ddPCR measurements (Table 2). RT-nested PCR yielded inconsistent results at low copy numbers. Some samples with very low or even undetectable copy numbers by RT-ddPCR showed positive nested PCR bands, while other samples with similar or higher copy numbers remained negative (Table 2). RT-ddPCR detected SFTSV RNA in 24 SFTS-positive samples with a sensitivity of 82.75%, while RT-qPCR identified 21 positive samples with a sensitivity of 72.4%, which was lower than that of RT-ddPCR (Fig. 2A). RT-nested PCR amplified positive bands in 25 SFTS-positive samples and showed a sensitivity of 86.21% (Fig. 2A). Despite the higher sensitivity of RT-nested PCR, the specificity was 89.66%, whereas RT-ddPCR showed 100% specificity (Fig. 2B). Therefore, lower specificity may have contributed to an overestimation of sensitivity and potentially interfered with the accurate and reliable diagnosis of SFTS patients with RT-nested PCR methods. To better assess diagnostic reliability, the overall accuracy was calculated. RT-ddPCR showed an accuracy of 91.38%, which was higher than both RT-qPCR (86.21%) and RT-nested PCR (87.93%). These results support RT-ddPCR as the most accurate diagnostic system considering both sensitivity and specificity.

We comprehensively evaluated the diagnostic performance of the three PCR assays using several diagnostic metrics (Fig. 2). ROC curves were generated and AUC values were calculated, which showed that RT-ddPCR had the highest diagnostic performance (AUC = 0.91) (Fig. 2C). The comprehensive evaluation of AUC, sensitivity, specificity, and accuracy is summarized in Fig. 2D, further demonstrating that RT-ddPCR is the most reliable diagnostic system for SFTS patients, with an AUC of 0.91, a sensitivity of 82.75%, a specificity of 100%, and an accuracy of 91.38%.

To investigate the factors underlying the false-negative results in conventional assays, we analyzed the duration between symptom onset and specimen collection (Table 2). We observed that conventional RT-qPCR and RT-nested PCR frequently yielded false-negative results in samples collected more than 10–12 days after symptom onset (e.g., sample nos. 4, 26, and 28). In these late-stage samples, viral clearance naturally leads to low viral RNA concentrations (range: 2.37–16.24 copies/μL as measured by RT-ddPCR), surpassing the analytical sensitivity of standard PCR methods. These findings indicate that the clinical sensitivity of molecular diagnosis is highly time dependent and that RT-ddPCR offers superior reliability for late-presenting patients. In addition, we investigated the potential causes of the false-positive results observed with nested RT-PCR. Further experiments using serum samples with and without spiked *O. tsutsugamushi* genomic DNA showed that our assay did not exhibit detectable cross-reactivity or non-specific amplification for *O. tsutsugamushi* (Fig. S10). These findings indicate that the three false-positive results observed with nested RT-PCR are more likely attributable to sporadic contamination events inherent to nested PCR rather than to cross-reactivity with other pathogens.

### Laboratory parameters strongly correlated with SFTSV RNA copies in early-stage SFTS patients

**Fig 3:**
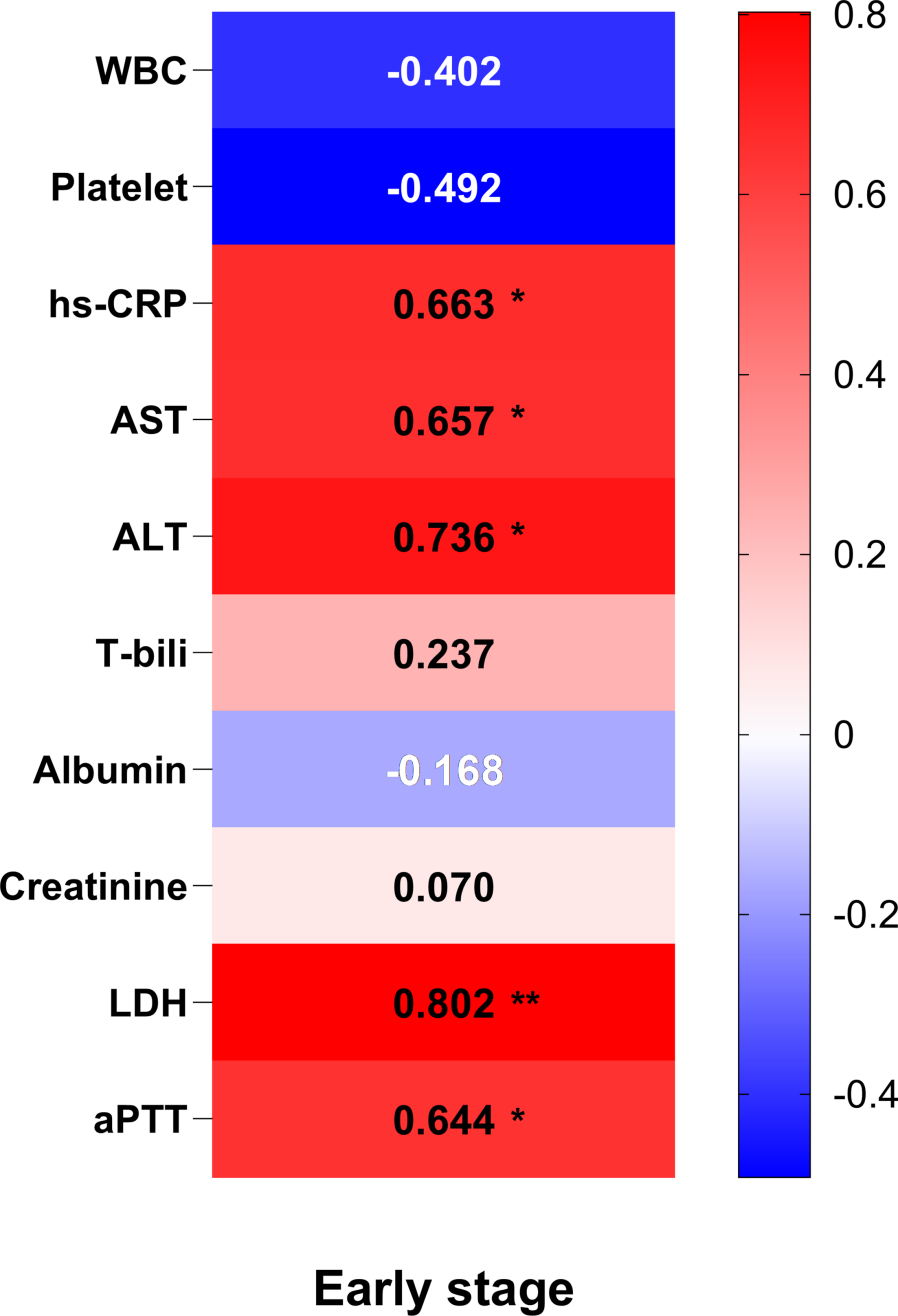
Spearman’s correlation heatmap between viral RNA loads quantified by RT-ddPCR and clinical parameters in early-stage SFTS patients. The heatmap illustrates Spearman correlation coefficients (*R*) between viral RNA loads quantified by RT-ddPCR and clinical parameters in early-stage SFTS patients, defined as those with symptom onset within 10 days and laboratory tests performed within 3 days of admission. Positive correlations are shown in red, and negative correlations are shown in blue, with different color intensities representing the strength of the correlation. Significant correlations (*P* < 0.05) are marked with an asterisk (*), and correlations with *P* < 0.01 are marked with double asterisks (**). Albumin denotes serum albumin; creatinine denotes serum creatinine, and "platelet" denotes platelet count. ALT, alanine aminotransferase; AST, aspartate aminotransferase; aPTT, activated partial thromboplastin time; hs-CRP, high-sensitivity C-reactive protein; LDH, lactate dehydrogenase; T-bili, total bilirubin; WBC, white blood cell count.

The correlation between viral RNA loads and key laboratory parameters was analyzed in early-stage SFTS patients, defined as those with symptom onset within 10 days and laboratory tests performed within 3 days of admission (Fig. 3). In this group, significant positive correlations were found for hs-CRP (*r* = 0.6626, *P* = 0.0418), aspartate aminotransferase (AST) (*r* = 0.6565, *P* = 0.0448), alanine aminotransferase (ALT) (*r* = 0.7356, *P* = 0.0190), LDH (*r* = 0.8024, *P* = 0.0075), and activated partial thromboplastin time (aPTT) (*r* = 0.6444, *P* = 0.05) with SFTSV RNA copy numbers (Fig. 3). These findings confirm that hs-CRP, AST, ALT, LDH and aPTT are all strongly associated with high SFTSV RNA copy numbers in early-stage SFTS patients.

Although WBC count (*r* = −0.402, *P* = 0.2467) and platelet count (*r* = −0.492, *P* = 0.1503) did not show statistically significant correlations, they showed moderate negative correlations. This suggests a trend of decreasing values as viral RNA loads increase. Additionally, total bilirubin (*r* = 0.237, *P* = 0.5060), creatinine (*r* = 0.070, *P* = 0.8456), and albumin (*r* = −0.168, *P* = 0.6381) exhibited weak correlations with SFTSV RNA copy numbers, suggesting minimal associations with viral load.

## DISCUSSION

The ddPCR system partitions a reaction mixture into tens of thousands of nanoliter-sized droplets, each of which serves as an independent reaction unit (18). This partitioning increases the local template concentrations within each droplet and disperses the impact of PCR inhibitors present in complex sample matrices such as human sera. The partitioning steps allow for reliable amplification and sensitive detection of pathogen nucleic acids via ddPCR even at low concentrations (14, 19). In addition, ddPCR provides absolute quantification of target molecules without the need for external standard curves by counting the number of positive droplets after amplification (20, 21). Therefore, we investigated whether RT-ddPCR has potential for improving molecular and clinical diagnosis of SFTS by comparing its diagnostic performance directly with RT-qPCR and RT-nested PCR.

The diagnostic performance of the three PCR methods was quantitatively compared first with RNA samples extracted from human serum reference materials spiked with SFTSV nucleic acid reference materials (22). The SFTSV nucleic acid reference material used in this study is a metrologically traceable reference material encapsulated in replication-incompetent virus-like particles (VLPs) that are produced at the Korea Research Institute of Standards and Science. In brief, lentivirus-based VLPs containing modified SFTSV L, M, and S segment RNAs derived from genotype A were produced by co-transfecting HEK293T/17 cells with a self-inactivating lentiviral transfer plasmid that encoded the respective SFTSV segment and a packaging plasmid that provided Gag, Pol, and Rev. The assigned copy numbers were determined by absolute quantification using droplet digital PCR. These reference materials provide traceable quantitative values and uncertainty characteristics, thus ensuring the reproducibility and reliability of the test results (23).

Our results demonstrated that RT-ddPCR showed the highest analytical sensitivity among the three PCR methods (Fig. 1). This was reflected in the diagnosis of actual patient samples as well, where RT-ddPCR had higher sensitivity than RT-qPCR and higher specificity than RT-nested PCR. Overall, RT-ddPCR showed the most accuracy and the highest AUC among the three PCR methods (Fig. 2). Considering that viral loads may be low in complex clinical specimens during the early phase of SFTS infection, our results suggest that RT-ddPCR could enhance the molecular diagnosis of SFTS over existing PCR-based methods (24). The addition of temporal data further validates the clinical utility of RT-ddPCR. While conventional assays are often sufficient during the peak viremic phase, their performance diminishes as viral clearance begins. Our results show that RT-ddPCR can detect low-abundance viral RNA even up to 25 days post-symptom onset (sample no. 26), a period during which conventional RT-qPCR and RT-nested PCR often fail. This enhanced sensitivity is crucial for accurate epidemiological surveillance and for managing patients who seek medical attention during the subacute or convalescent phases of SFTS. In actual clinical practice, differential diagnosis is essential, and scrub typhus is a mite-borne disease requiring differential diagnosis from SFTS in endemic regions. Therefore, inclusion of scrub typhus patient samples as a negative control further validated the specificity and accuracy offered by RT-ddPCR, which provided higher confidence in the molecular diagnosis of SFTS.

The RT-ddPCR system was also investigated for its applicability in improving clinical diagnosis based on absolute quantification capability. Our results demonstrated significant positive correlations between viral RNA copies and hs-CRP, AST, ALT, LDH, and aPTT and a negative correlation with white blood cells and platelet counts from patients in the early-infection stage (Fig. 3). This was consistent with previous reports that determined disease severity and mortality in relation to viral loads in SFTS patients (25–27). Unlike previous studies, we re-grouped and focused patients into early-infection stage, which showed higher positive correlations in AST, ALT, LDH, and hs-CRP compared to previous reports without such classifications (28, 29). This suggests that focusing on the early stage of the disease reveals more direct associations between viral load and clinical parameters compared to analyses conducted over the entire disease course. Furthermore, our results also showed stronger correlations with AST and LDH against qPCR-based methods (30), which is a result of higher sensitivity and improved diagnostic performance of RT-ddPCR. In summary, RT-ddPCR enables early risk stratification based on absolute viral load and related clinical markers from patients with suspected or early-stage SFTS.

In conclusion, this study demonstrated that RT-ddPCR improves analytical sensitivity and diagnostic performance for molecular diagnosis of SFTS compared with conventional PCR methods. Importantly, the improved specificity of RT-ddPCR was validated against scrub typhus, a major differential diagnosis of SFTS in endemic regions, highlighting its clinical utility in real-world diagnostic settings. However, we acknowledge it as a limitation of this study that the negative control group consisted exclusively of patients with scrub typhus; thus, the diagnostic specificity was evaluated against only a single alternative diagnosis. These findings support RT-ddPCR as a useful quantitative tool to guide clinical decision-making in patients with suspected or confirmed SFTS.

## SUPPLEMENTAL MATERIAL

Supplemental materials jcm.00367-26-s0001.docx Fig. S1 to S10, Table S1, and Supplemental methods.

ASM does not own the copyrights to Supplemental Material that may be linked to, or accessed through, an article. The authors have granted ASM a non-exclusive, world-wide license to publish the Supplemental Material files. Please contact the corresponding author directly for reuse.

## References

[1] Casel MA, Park SJ, Choi YK. 2021. Severe fever with thrombocytopenia syndrome virus: emerging novel phlebovirus and their control strategy. Exp Mol Med 53:713–722. doi:10.1038/s12276-021-00610-1 33953322 PMC8178303

[2] Cui H, Shen S, Chen L, Fan Z, Wen Q, Xing Y, Wang Z, Zhang J, Chen J, La B, et al.. 2024. Global epidemiology of severe fever with thrombocytopenia syndrome virus in human and animals: a systematic review and meta-analysis. Lancet Reg Health West Pac 48:101133. doi:10.1016/j.lanwpc.2024.101133 39040038 PMC11261768

[3] Kim D, Lai C-J, Cha I, Jung JU. 2024. Current progress of severe fever with thrombocytopenia syndrome virus (SFTSV) vaccine development. Viruses 16:128. doi:10.3390/v16010128 38257828 PMC10818334

[4] Afzal A. 2020. Molecular diagnostic technologies for COVID-19: limitations and challenges. J Adv Res 26:149–159. doi:10.1016/j.jare.2020.08.002 32837738 PMC7406419

[5] Sun Y, Liang M, Qu J, Jin C, Zhang Q, Li J, Jiang X, Wang Q, Lu J, Gu W, et al.. 2012. Early diagnosis of novel SFTS bunyavirus infection by quantitative real-time RT-PCR assay. J Clin Virol 53:48–53. doi:10.1016/j.jcv.2011.09.031 22024488

[6] Tian W, Ren X, Gao X, Zhang Y, Chen Z, Zhang W. 2022. Accuracy of reverse-transcription polymerase chain reaction and loop-mediated isothermal amplification in diagnosing severe fever with thrombocytopenia syndrome: a systematic review and meta-analysis. J Med Virol 94:5922–5932. doi:10.1002/jmv.28068 35968756 PMC9804528

[7] Dutta D, Naiyer S, Mansuri S, Soni N, Singh V, Bhat KH, Singh N, Arora G, Mansuri MS. 2022. COVID-19 diagnosis: a comprehensive review of the RT-qPCR method for detection of SARS-CoV-2. Diagnostics (Basel) 12:1503. doi:10.3390/diagnostics12061503 35741313 PMC9221722

[8] Deepak S, Kottapalli K, Rakwal R, Oros G, Rangappa K, Iwahashi H, Masuo Y, Agrawal G. 2007. Real-time PCR: revolutionizing detection and expression analysis of genes. Curr Genomics 8:234–251. doi:10.2174/138920207781386960 18645596 PMC2430684

[9] Jalal S, Hwang SY, Kim C-M, Kim D-M, Yun NR, Seo J-W, Young Kim D, Jung SI, Kim UJ, Kim SE, et al.. 2021. Comparison of RT-PCR, RT-nested PCRs, and real-time PCR for diagnosis of severe fever with thrombocytopenia syndrome: a prospective study. Sci Rep 11:16764. doi:10.1038/s41598-021-96066-4 34408188 PMC8373928

[10] Kim C-M, Kim D-M, Yun N-R. 2021. Clinical usefulness of nested reverse-transcription polymerase chain reaction for the diagnosis of severe fever with thrombocytopenia syndrome. Am J Trop Med Hyg 105:999–1003. doi:10.4269/ajtmh.21-0183 34339382 PMC8592136

[11] Dong F. 2022. Molecular oncology diagnostics, an issue of the clinics in laboratory medicine. E-Book: Elsevier Health Sciences. 10.1016/j.cll.2022.06.001 36150827

[12] Ikewaki J, Ohtsuka E, Kawano R, Ogata M, Kikuchi H, Nasu M. 2003. Real-time PCR assay compared to nested PCR and antigenemia assays for detecting cytomegalovirus reactivation in adult t-cell leukemia-lymphoma patients. J Clin Microbiol 41:4382–4387. doi:10.1128/JCM.41.9.4382-4387.2003 12958273 PMC193823

[13] Taylor SC, Laperriere G, Germain H. 2017. Droplet digital PCR versus qPCR for gene expression analysis with low abundant targets: from variable nonsense to publication quality data. Sci Rep 7:2409. doi:10.1038/s41598-017-02217-x 28546538 PMC5445070

[14] Li H, Bai R, Zhao Z, Tao L, Ma M, Ji Z, Jian M, Ding Z, Dai X, Bao F, et al.. 2018. Application of droplet digital PCR to detect the pathogens of infectious diseases. Biosci Rep 38:BSR20181170. doi:10.1042/BSR20181170 30341241 PMC6240714

[15] Pinheiro LB, Coleman VA, Hindson CM, Herrmann J, Hindson BJ, Bhat S, Emslie KR. 2012. Evaluation of a droplet digital polymerase chain reaction format for DNA copy number quantification. Anal Chem 84:1003–1011. doi:10.1021/ac202578x 22122760 PMC3260738

[16] Mahendran P, Liew JWK, Amir A, Ching X-T, Lau Y-L. 2023. Correction: droplet digital polymerase chain reaction (ddPCR) for the detection of *Plasmodium knowlesi* and *Plasmodium vivax*. Malar J 22:324. doi:10.1186/s12936-023-04733-w 37880730 PMC10601243

[17] Hwang J-H, Kim J, Sun IO, Lee TH, Chung KM, Lee C-S. 2022. New genotypes and diversity of *Orientia tsutsugamushi* DNA samples from patients with scrub typhus in South Korea as determined by multilocus sequence typing. Am J Trop Med Hyg 107:420–426. doi:10.4269/ajtmh.21-0850 35895396 PMC9393445

[18] Hindson BJ, Ness KD, Masquelier DA, Belgrader P, Heredia NJ, Makarewicz AJ, Bright IJ, Lucero MY, Hiddessen AL, Legler TC, et al.. 2011. High-throughput droplet digital PCR system for absolute quantitation of DNA copy number. Anal Chem 83:8604–8610. doi:10.1021/ac202028g 22035192 PMC3216358

[19] Váňová B, Malicherova B, Burjanivová T, Liskova A, Janikova K, Jasek K, Lasabová Z, Tatár M, Plank L. 2021. Droplet digital PCR as a novel dia-gnostic tool. Klin Onkol 34:33–39. doi:10.48095/ccko202133 33657817

[20] Quan P-L, Sauzade M, Brouzes E. 2018. dPCR: a technology review. Sensors (Basel) 18:1271. doi:10.3390/s18041271 29677144 PMC5948698

[21] Kuypers J, Jerome KR. 2017. Applications of digital PCR for clinical microbiology. J Clin Microbiol 55:1621–1628. doi:10.1128/JCM.00211-17 28298452 PMC5442518

[22] Roh Y, Baek Y, Kim MG, Koo B, Seo R, Lee J, Jung S, Kim SK, Bae M, Lee C-S, et al.. 2025. Semi-automated diagnostic system and severe fever with thrombocytopenia syndrome (SFTS) viral RNA reference materials encapsulated in virus-like particles for on-site diagnosis of SFTS. Sensors and Actuators B: Chemical 433:137530. doi:10.1016/j.snb.2025.137530

[23] Hayden RT. 2023. Primary quantitative reference standards for viral nucleic acids should be developed using digital polymerase chain reaction instead of consensus testing. J Clin Microbiol 61:e0133822. doi:10.1128/jcm.01338-22 36475837 PMC9879095

[24] Yu Y, Li J, Liu Q, Zhai R, Dai Y, Sun L. 2025. Advances in research on severe fever with thrombocytopenia syndrome virus. Front Microbiol 16:1622394. doi:10.3389/fmicb.2025.1622394 40771693 PMC12325320

[25] Cui N, Bao X-L, Yang Z-D, Lu Q-B, Hu C-Y, Wang L-Y, Wang B-J, Wang H-Y, Liu K, Yuan C, et al.. 2014. Clinical progression and predictors of death in patients with severe fever with thrombocytopenia syndrome in China. J Clin Virol 59:12–17. doi:10.1016/j.jcv.2013.10.024 24257109

[26] Hao Y, Sun Y, Pan Z, Sun J, Liang M, Zhang A, Yang J. 2025. Establishment and application of a grading diagnostic model for early detection of severe fever with thrombocytopenia syndrome. Ann Med 57:2493307. doi:10.1080/07853890.2025.2493307 40254938 PMC12013140

[27] Xu X, Sun Z, Liu J, Zhang J, Liu T, Mu X, Jiang M. 2018. Analysis of clinical features and early warning indicators of death from severe fever with thrombocytopenia syndrome. Int J Infect Dis 73:43–48. doi:10.1016/j.ijid.2018.05.013 29859247

[28] Zhang Y, Tian W, Zhang S, Lin L, Song C, Liu Y, Xu Y, Zhang L, Geng S, Li X, et al.. 2025. Enhancing sensitivity in detecting severe fever with thrombocytopenia syndrome virus: development of a reverse transcription-droplet digital polymerase chain reaction. J Infect Dis 231:512–520. doi:10.1093/infdis/jiae442 39352170

[29] Gao M, Zhao L, Dong Q, Zhang X, Li L, Zhao D, Zhou Q, Xu Y, Zhen P, Lu S, et al.. 2025. Development and evaluation of the digital PCR-based method for clinical monitoring of viral loads during severe fever with thrombocytopenia syndrome virus infection. J Clin Virol 177:105777. doi:10.1016/j.jcv.2025.105777 40068230

[30] Yang Z-D, Hu J-G, Lu Q-B, Guo C-T, Cui N, Peng W, Wang L-Y, Qin S-L, Wang H-Y, Zhang P-H, et al.. 2016. The prospective evaluation of viral loads in patients with severe fever with thrombocytopenia syndrome. J Clin Virol 78:123–128. doi:10.1016/j.jcv.2016.03.017 27062673

