# Supplementary material for: Droplet digital PCR-based nucleic acid amplification test for improved molecular and clinical diagnosis of severe fever with thrombocytopenia syndrome patients
Source: J Clin Microbiol. 2026 Jul 27;64(8):e00367-26. doi: 10.1128/jcm.00367-26 (PMC13463810; doi:10.1128/jcm.00367-26)
Supplement: Supplementary file 1 [file jcm.00367-26-s0001.docx]

**Supplemental Materials**

**Droplet digital PCR-based nucleic acid amplification test for improved molecular and clinical diagnosis of Severe Fever with Thrombocytopenia Syndrome patients**

**Yujin Baek^1,2^, Minchae Koo^1,3^, Yong Shin^2^, Chang-Seop Lee^4,5*^, Il-Hwan Kim^1,6**^**

^1^Division of Biomedical Metrology, Korea Research Institute of Standards and Science, Daejeon, 34113, Republic of Korea; ^2^Department of Biotechnology, College of Life Science and Biotechnology, Yonsei University, Seoul, 03722, Republic of Korea; ^3^Department of Biological Sciences, Korea Advanced Institute of Science and Technology, Daejeon 34141, Republic of Korea; ^4^Department of Internal Medicine, Jeonbuk National University Medical School, Jeonju, 54896, Republic of Korea; ^5^Research Institute of Clinical Medicine of Jeonbuk National University-Biomedical Research Institute of Jeonbuk National University Hospital, Jeonju, 54907, Republic of Korea; ^6^Precision Measurement Science, University of Science and Technology, Daejeon, Republic of Korea.

*Corresponding author: Chang-Seop Lee, MD/PhD

E-mail: [lcsmd@jbnu.ac.kr](mailto:lcsmd@jbnu.ac.kr)

**Corresponding author: Il-Hwan Kim, PhD

E-mail addresses: [ihkim@kriss.re.kr](mailto:ihkim@kriss.re.kr)

**Contents**

**Supplementary Methods**

**Table S1.** Primer and probe sequences used in this study.

**Figure S1.** Multiple sequence alignment of the SFTSV M segment region targeted by the in-house universal assay across genotypes A–F.

**Figure S2.** Analytical performance of the in-house universal assay targeting the SFTSV M segment for genotypes A–F across comparable dilution ranges.

**Figure S3.** Representative 1D-amplitude plots for RT-ddPCR showing target specific amplification of SFTSV genotypes A-F using universal primer-probe set.

**Figure S4.** Representative RT-qPCR amplification curves showing target specific amplification of SFTSV genotypes A-F using universal primer-probe set.

**Figure S5.** Amplification products of RT-nested PCR showing target specific amplification of SFTSV genotypes A-F using universal primer sets.

**Figure S6.** Representative detection readouts near the detection limits of RT‑ddPCR, RT‑qPCR and RT‑nested PCR.

**Figure S7.** Representative 1D-amplitude plots of RT-ddPCR for SFTSV RNA detection in SFTS and febrile patient sera.

**Figure S8.** Representative RT-qPCR amplification curves for SFTSV RNA detection in SFTS and febrile patient sera.

**Figure S9.** RT-nested PCR amplification products for SFTSV RNA detection in SFTS and febrile patient sera.

**Figure S10.** Specificity evaluation of the in-house assay targeting SFTSV M segment in the presence of *Orientia tsutsugamushi* DNA.

**Supplementary Methods**

**Viral RNA extraction from SFTSV nucleic acid reference materials**

To extract RNA under conditions similar to actual clinical samples, human serum reference material was spiked with SFTSV nucleic acid reference material encapsulated in virus-like particles. The spiked solution was serially diluted from 1.27 × 10^5^ to 1.27 × 10^-1^ copies/µL, and viral RNA was extracted from 100 μL of each diluted solution using QIAamp Viral RNA Mini Kit (QIAGEN, Germany), which is a commercially available kit widely used for viral RNA extraction from serum or plasma in clinical and research settings (1, 2). The extracted RNA was used for comparative analysis of RT-ddPCR, RT-qPCR and RT-nested PCR.

**Viral RNA extraction from SFTS patient sera and scrub typhus patient samples**

Viral RNA was extracted from sera of 29 patients with confirmed SFTS and 29 patients with scrub typhus using the QIAamp Viral RNA Mini Kit (QIAGEN, Germany), according to the manufacturer's instructions. Powercheck SFTSV Real-time PCR kit (KogenBiotech, Korea) was used to determine the genotype of SFTSV isolated from patient samples.

**Design of the in-house universal primer and probe for detection of** **SFTSV M segment gene**

For RT-ddPCR and RT-qPCR, a universal primer and probe set targeting the M segment was designed for the purpose of detecting all SFTSV genotypes, from A to F (Table S1). The probe was labeled with the fluorescent dye 6-carboxyfluorescein (FAM) at the 5' end and the quencher Black Hole Quencher 1 (BHQ1) at the 3' end. Primers for RT-nested PCR were also designed for detection of SFTSV pan-genotypically (Table S1). The first PCR primer set we used was modified from primers used in previous studies (3). The primer set previously described by Han et al (4) was used as the second PCR primer set in this study. To verify that the in‑house assays could detect all known SFTSV genotypes, genotype‑defined RNA materials were used for their evaluation. Viral RNA of genotype A was derived from the SFTSV nucleic acid reference material used in this study, whereas genotypes B–E were pathogen‑derived RNAs obtained from the National Culture Collection for Pathogens (NCCP), Republic of Korea. Genotype F RNA was obtained from the Korea Zoonosis Research Institute at Jeonbuk National University, where it had been genotyped and confirmed as genotype F.

**Reverse transcription-quantitative PCR experiments**

RT-qPCR was performed using the One Step PrimeScript III RT-PCR Kit (TaKaRa, Japan), and Ct values were obtained for viral RNA extracted from SFTSV nucleic acid reference materials and patient sera. The reaction mixture was prepared in a total volume of 20 μL and contained 333 nM of each primer, 167 nM of probe and 2 μL of extracted RNA as template, according to the manufacturer’s instructions. qPCR analysis was carried out using the Bio-Rad CFX96 Real-Time PCR System (Bio-Rad Laboratories, USA). A fixed fluorescence threshold was first predefined during the LoD experiments using reference materials, and this same threshold was then applied uniformly to all subsequent runs with clinical samples. Ct values were determined using this predefined threshold level.

**Reverse transcription-droplet digital PCR experiments**

RT-ddPCR was performed using the One-Step RT-ddPCR Advanced Kit for (Bio-Rad Laboratories, USA) on the same viral RNA samples used in RT-qPCR. Each reaction mixture was prepared in a total volume of 22 μL and contained 303 nM of each primer, 152 nM of probe and 2 μL of extracted RNA as template, with all other components prepared according to the manufacturer’s protocol. Automated Droplet Generator (Bio-Rad Laboratories, USA) was used to generate the droplets. The amplified droplets were analyzed using QX200 Droplet Reader (Bio-Rad Laboratories, USA) and QuantaSoft software version 1.7.4 (Bio-Rad Laboratories, USA). All thresholds were set manually to distinguish between positive and negative droplets.

**Nested PCR experiments**

The first PCR was performed using the SuperScript™ III One-Step RT-PCR System with Platinum™ Taq DNA Polymerase (Thermo Fisher Scientific, Lithuania) in a total reaction volume of 20 µL, following the manufacturer's instructions. The reaction mixture was prepared according to the manufacturer's instructions and contained 300 nM forward and reverse primers and 2 μL of extracted RNA as template for the first PCR. For the second PCR, the One-Step PrimeScript III RT-PCR Kit (TaKaRa, Japan) was used with 20 µL of reaction volume, including the first-round PCR product as the template and 300 nM of forward and reverse primers. The final PCR product was analyzed by electrophoresis on a 1% agarose gel.

**Determination of limit of detection and cut‑off values**

SFTSV nucleic acid reference material at concentrations ranging from 1.27 × 10^5^ to 1.27 × 10^-1^ copies/µL was tested by RT‑ddPCR, RT‑qPCR and RT‑nested PCR to evaluate the analytical performance of the three assays. Each concentration was analyzed in eight replicate reactions. The limit of detection was estimated by probit regression analysis, using the proportion of positive results at each concentration to calculate the RNA concentration corresponding to a 95% detection probability with 95% confidence intervals.

For RT-ddPCR, samples were classified as positive when at least two positive droplets were observed, corresponding to an estimated concentration of approximately 4.3–4.7 copies/µL. For RT-qPCR, a Ct value of 35 was selected as the positivity cutoff based on the observation that the mean Ct value at the lowest concentration consistently detected in all 8 of 8 replicate reactions was 34.65. For the nested RT-PCR assay, samples were considered positive when the specific 560 bp amplification product was observed under the defined reaction conditions and negative when no such product was detected.

**Evaluation of diagnostic performance metrics**

Sensitivity, specificity, and accuracy were calculated based on the number of true positives (TP), true negatives (TN), false positives (FP), and false negatives (FN). Sensitivity is defined as the proportion of true positives among all patients with the condition, calculated as TP/(TP + FN)%. Specificity is defined as the proportion of true negatives among all subjects without the disease, calculated as TN/(TN + FP)%. Accuracy is calculated as the proportion of correctly classified samples (both true positives and true negatives) among all samples, and is calculated as (TP + TN)/(TP + TN + FP + FN)%. Diagnostic performance was evaluated by generating receiver operating characteristic (ROC) curves using the sensitivity and specificity values at specific thresholds. The area under the curve (AUC) was calculated to quantify the overall diagnostic performance of each method. AUC values close to 1 indicate excellent diagnostic ability, while values near 0.5 suggest random performance.

**Assessment of the correlation between viral load and laboratory parameters**

Laboratory parameters analyzed in SFTS patients included white blood cell (WBC) count, platelet count, high-sensitivity C-reactive protein (hs-CRP), aspartate aminotransferase (AST), alanine aminotransferase (ALT), total bilirubin (T-bili), serum albumin, serum creatinine, lactate dehydrogenase (LD) and activated partial thromboplastin time (aPTT). The correlation between viral load and laboratory parameter was assessed by Spearman's rank test for three stages.

**References**

1.	Yao E, Tavis JE, edu V-CSGts. A general method for nested RT-PCR amplification and sequencing the complete HCV genotype 1 open reading frame. Virology journal. 2005;2(1):88.

2.	Natarajan A, Han A, Zlitni S, Brooks EF, Vance SE, Wolfe M, et al. Standardized and optimized preservation, extraction and quantification techniques for detection of fecal SARS-CoV-2 RNA. medRxiv. 2021.

3.	Kim C-M, Kim D-M, Yun N-R. Clinical Usefulness of Nested Reverse-Transcription Polymerase Chain Reaction for the Diagnosis of Severe Fever with Thrombocytopenia Syndrome. The American Journal of Tropical Medicine and Hygiene. 2021;105(4):999.

4.	Yun S-M, Lee W-G, Ryou J, Yang S-C, Park S-W, Roh JY, et al. Severe fever with thrombocytopenia syndrome virus in ticks collected from humans, South Korea, 2013. Emerging infectious diseases. 2014;20(8):1358.

**Table S1.** Primer and probe sequences used in this study.

| **Name** | **Sequences (5'-3')** | **Mer** |
| --- | --- | --- |
| SFTSV universal M FW | CATCATGAGGGAGCACAAGAC | 21 |
| SFTSV universal M RV | TCCCCATGTTCACAACCTGC | 20 |
| SFTSV universal M P | [FAM] TGCAAGACCAGTGGATCATGCAGGG [BHQ1] | 25 |
| 1^st^ nested PCR M FW | TCATCCTGACYTATTYTGCAATWG | 25 |
| 1^st^ nested PCR M RV | TAAGTYACACTCACACCCTTGAA | 23 |
| 2^nd^ nested PCR M FW | GATGAGATGGTCCATGCTGATTCTAA | 26 |
| 2^nd^ nested PCR M RV | CTCATGGGGTGGAATGTCCTCAC | 23 |

**Figure S1.** Multiple sequence alignment of the SFTSV M segment region targeted by the in-house universal assays across genotypes A–F. The alignment shows the consensus sequence and representative sequences for each genotype, with the binding sites of the forward primer, probe and reverse primer indicated by red boxes and arrows.

**Figure S2.** Analytical performance of the in-house universal assay targeting the SFTSV M segment for genotypes A–F across comparable dilution ranges. (A) Representative 1D-amplitude plots of RT-ddPCR results obtained from 10-fold serial dilutions of RNA corresponding to SFTSV genotypes A–F, including no‑template controls (NTCs). (B) Linear regression lines for each genotype generated from three dilution levels, plotting log_10_ RNA copies/µL against the dilution factor.

**Figure S3.** Representative 1D-amplitude plots for RT-ddPCR showing target specific amplification of SFTSV genotypes A-F using the universal primer-probe set. (A) 1D-amplitude plot of SFTSV genotype A. (B) 1D-amplitude plot of SFTSV genotype B. (C) 1D-amplitude plot of SFTSV genotype C. (D) 1D-amplitude plot of SFTSV genotype D. (E) 1D-amplitude plot of SFTSV genotype E. (F) 1D-amplitude plot of SFTSV genotype F. NTC, No Template Control.

**Figure S4.** Representative RT-qPCR amplification curves showing target specific amplification of SFTSV genotypes A-F using universal primer-probe set. (A) Amplification curve for SFTSV genotype A. (B) Amplification curve for SFTSV genotype B. (C) Amplification curve for SFTSV genotype C. (D) Amplification curve for SFTSV genotype D. (E) Amplification curve for SFTSV genotype E. (F) Amplification curve for SFTSV genotype F. The *x*-axis represents the number of cycles and the *y*-axis represents the relative fluorescence units (RFU).

**Figure S5.** Amplification products of RT-nested PCR showing target specific amplification of SFTSV genotypes A-F using universal primer sets. (A) First PCR amplification product of 643 bp for SFTSV genotypes A-F. (B) Second PCR amplification product of 560 bp for SFTSV genotypes A-F. A, SFTSV genotype A; B, SFTSV genotype B, C, SFTSV genotype C; D, SFTSV genotype D; E, SFTSV genotype E; F, SFTSV genotype F; N, No Template Control.

**Figure S6.** Representative detection readouts near the detection limits of RT‑ddPCR, RT‑qPCR and RT‑nested PCR. (A) 1D-amplitude plot of RT‑ddPCR using SFTSV nucleic acid reference material at 12.7, 1.27, and 0.127 copies/µL (10^-5^, 10^-6^, 10^-7^), with eight replicate reactions displayed consecutively for each concentration. (B) Amplification curves of RT‑qPCR generated from the low‑concentration range (e.g., 127, 12.7, and 1.27 copies/µL; 10^-4^, 10^-5^, 10^-6^). (C) Amplification products of RT‑nested PCR obtained from eight replicate reactions at 12.7, 1.27, and 0.127 copies/µL (10^-5^, 10^-6^, 10^-7^).

**Figure S7.** Representative 1D-amplitude plots of RT-ddPCR for SFTSV RNA detection in SFTS and febrile patient sera. (A) 1D-amplitude plots for SFTSV RNA in SFTS patient sera. (B) 1D-amplitude plots for SFTSV RNA in febrile patient sera.

**Figure S8.** Representative RT-qPCR amplification curves for SFTSV RNA detection in SFTS and febrile patient sera. (A) Amplification curves for SFTSV RNA in SFTS patient sera. (B) Amplification curves for SFTSV RNA in febrile patient sera. The *x*-axis represents the number of cycles and the *y*-axis represents the relative fluorescence units (RFU).

**Figure S9.** RT-nested PCR amplification products for SFTSV RNA detection in SFTS and febrile patient sera. (A) Agarose gel electrophoresis results of RT-nested PCR amplification for SFTSV RNA in SFTS patient sera. (B) Agarose gel electrophoresis results of RT-nested PCR amplification for SFTSV RNA in febrile patient sera. The band at 560 bp corresponds to the amplified product. NTC, No Template Control.

**Figure S10.** Specificity evaluation of the in-house assay targeting SFTSV M segment in the presence of *Orientia tsutsugamushi* DNA. Lanes correspond to SFTSV‑negative serum RNA without *O. tsutsugamushi* genomic DNA (SFTSV−/OT−), SFTSV‑negative serum RNA spiked with *O. tsutsugamushi* genomic DNA (SFTSV−/OT+), SFTSV‑positive serum RNA without *O. tsutsugamushi* genomic DNA (SFTSV+/OT−), and SFTSV‑positive serum RNA spiked with *O. tsutsugamushi* genomic DNA (SFTSV+/OT+), and a no‑template control (NTC).
